# Theoretical Modeling Techniques and Their Impact on Tumor Immunology

**DOI:** 10.1155/2010/271794

**Published:** 2010-12-23

**Authors:** Anna Lena Woelke, Manuela S. Murgueitio, Robert Preissner

**Affiliations:** Structural Bioinformatics Group, Institute for Physiology, Charité - Universitätsmedizin Berlin, Lindenberger Weg 80, 13125 Berlin, Germany

## Abstract

Currently, cancer is one of the leading causes of death in industrial nations. While conventional cancer treatment usually results in the patient suffering from severe side effects, immunotherapy is a promising alternative. Nevertheless, some questions remain unanswered with regard to using immunotherapy to treat cancer hindering it from being widely established. To help rectify this deficit in knowledge, experimental data, accumulated from a huge number of different studies, can be integrated into theoretical models of the tumor-immune system interaction. Many complex mechanisms in immunology and oncology cannot be measured in experiments, but can be analyzed by mathematical simulations. Using theoretical modeling techniques, general principles of tumor-immune system interactions can be explored and clinical treatment schedules optimized to lower both tumor burden and side effects. In this paper, we aim to explain the main mathematical and computational modeling techniques used in tumor immunology to experimental researchers and clinicians. In addition, we review relevant published work and provide an overview of its impact to the field.

## 1. Introduction

Biological systems possess a high degree of complexity. The role a component plays in the organism is not only defined by its function but also by its interaction network [1]. The field of systems biology is concerned with that topic and aims at understanding the interactions between various components of the living cell, such as genes, proteins, and metabolites [2]. A huge mass of biological facts has been uncovered by molecular biology, but understanding biological complexity on a systems level can only be achieved by a combination of experimental and computational approaches [3]. 

The immune response to tumor formation represents a complex system that can solely be understood by using several different research strategies. In recent time, it has been shown that the immune system plays a pivotal role in the regulation of cancer, enhancing its growth by certain mechanisms [4, 5] and being capable of recognizing and eradicating tumors as well [5, 6]. As conventional cancer therapy usually involves severe side effects, increased research efforts have been made in order to stimulate the immune response against the tumor [7, 8]. Various approaches including vaccination [9, 10] or direct injection of antibodies [11], lymphocytes [12–14], or cytokines [15, 16] have been developed. 

**Figure 1:**
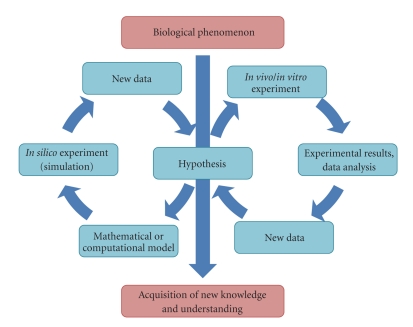
Steps in knowledge generation in system biology. Knowledge on a given biological phenomenon is generated in a stepwise manner by combining modeling and experimental techniques. A model is built up enabling the researcher to perform *in silico* experiments in order to predict the behavior of the biological system under given conditions. These predictions have, in turn, to be validated via *in vivo* or *in vitro* systems, leading to further refinement of the model and the underlying hypothesis.

Complex systems can be analyzed through several mathematical and computational approaches. An overview of the distinct steps in model generation is given in Figure 1. The available knowledge about a given biological phenomenon is used to build up a model. Once validated through the comparison with experimental results or the literature, the model can be used to perform *in silico* experiments, which allow the generation of hypotheses on the behavior of the biological system. These findings can in turn be verified *in vivo* or *in vitro* and the data acquired this way can be used to refine the model, hence enhancing its significance. This iterative course of action leads to a better understanding of biological systems. In this work, we introduce the basic steps in model generation and present the two most common methods in simulating the interaction between a growing tumor and the immune system: differential equations and rule-based models. We compare them and point out their impact on the field of tumor immunology in diverse applications as for example the identification of cancer mechanisms or tumor therapy.

## 2. Construction of a Mathematical Model

Constructing a model always starts with the collection of relevant data in order to define the problem. Afterwards, the model is built in an iterative process including parameter fitting and validation steps. In the next paragraph, we provide an overview of what kind of information is collected and how it is built into the network. We will then go on to explain how the model is refined and optimized.

### 2.1. Data Retrieval

The first step in model construction is the retrieval of information about the given parameters, for example, cell division rates or enzyme kinetics, which can be extracted from the literature or from public databases, for example KEGG [17] or Reactome [18] (see Table 1). Focused immunological information can be found in specialized databases (e.g. IEDB [19]). More detailed information about specific processes is extracted from experimental results. These can be gained by carrying out own experiments or cooperation with wet-lab researchers, or by carefully mining the published literature. In some cases, parameters are not experimentally accessible; here, data fitting must be performed to estimate them. The values must be adjusted so that the biological feasibility of the model is preserved (for a detailed review, see [20]).

### 2.2. Building and Refining the Model

The model is built and refined in an iterative process. The basic assumptions of the behavior of the system are formulated as differential equations or as computational rules and the parameters are defined as described above. The resulting model is used to simulate the system under different conditions and the results are compared to wet-lab experiments or clinical data. If the simulation differs from the experimental results, the model parameters get adjusted, or the model is refined to account for a more divergent systems behavior. In turn, the model can explain the system more in detail and gives more realistic predictions.

A model relating to the nuclear factor “kappa-light-chain-enhancer” of activated B-cells (NF-*κ*B) signaling pathway illustrates clearly how a mathematical model is refined in a stepwise manner by the addition of new experimental knowledge which in turn leads to an ever growing understanding of the system. Lipniacki et al. developed a mathematical model of the NF-*κ*B regulatory module [23]. The model exhibits two-compartment kinetics built as a system of differential equations and it can reproduce the time behavior of the involved protein and mRNA levels and of the catalytic activity of I*κ*B kinase. After further research, this model was refined to include two classes of switches that are invoked stochastically, namely, the cell-surface receptor activation by the tumor necrosis factor-*α* (TNF-*α*) ligand and the activation of genes by NF-*κ*B [24]. These stochastic switches allow single cells to respond differently to their neighbors, with each individual response being unequivocal. In cooperation with a wet-lab research group, it was shown that the activation by TNF-*α* is indeed heterogeneous and single cells respond in a digital process with fewer cells responding at lower doses [25]. In addition, they found that some parameters change analogously, for example, NF-*κ*B peak intensity, response time, and number of oscillations. Consequently, the mathematical model was refined again with a cellular variation in the amount of  TNF*α*-receptor and a nonlinear activation profile of I*κ*B kinase. This final model is able to reproduce both the digital and analogue dynamics as well as most gene expression profiles at all measured conditions. It can also predict the fraction of cells responding to consecutive short pulses of low-dose TNF-*α* with high accuracy. Our understanding of the TNF-*α*-induced NF-*κ*B-signaling has improved significantly through the close cooperation and reciprocal influence of experimental and theoretical research.

## 3. Differential Equations Systems

**Figure 2:**
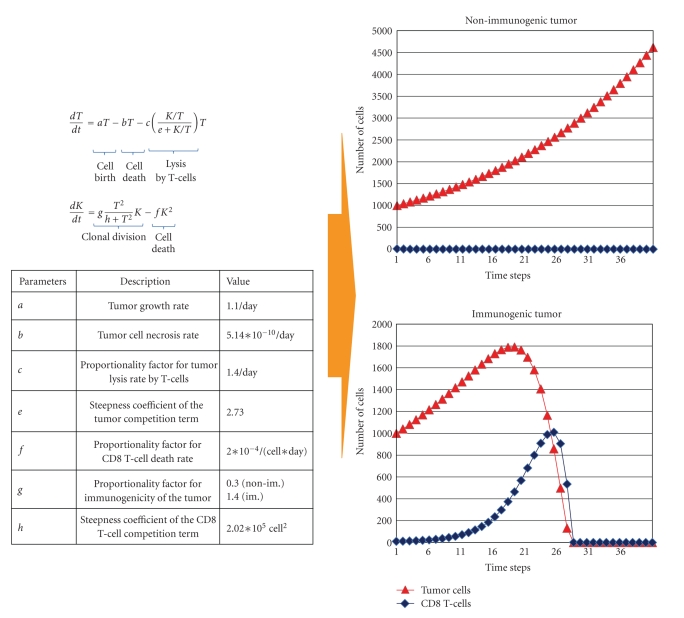
Differential equations system. A simplified differential equations system describes the interaction between tumor cells (*T*) and CD8 T-cells (*K*). The only difference between the simulation of a nonimmunogenic tumor (upper graph) and an immunogenic tumor (lower graph) is that parameter *g* is increased leading to an increase in tumor immunogenicity. Lysis of tumor cells by the CD8 T-cells and the activation of CD8 T-cells by tumor cells follow a Michaelis-Menten kinetic to account for saturation at high cell numbers. The death rate of CD8 T-cells is proportional to the square of the cell number to assure for fast declining waves of T-cell expansion. Equations and parameters modified from [27], parameters originally obtained from a published mouse study [28].

Differential equations are used to describe several principles in physics or chemistry or to simulate complex systems in biology and economics. A system of differential equations allows modeling of time-dependent cellular phenomena such as individual biochemical reactions, signal transduction cascades, or even the interaction between whole cell populations [26]. Each entity that is considered to be important for the question of interest is modeled by one differential equation describing its production and its decay or its influx and efflux from a compartment. In Figure 2, a simplified model is shown consisting of two equations describing the amount of tumor and CD8 T-cells over time. By changing only one parameter, the result can switch from an exponentially growing tumor to a tumor being recognized and destroyed by the immune system. An equivalent system of differential equations can be analyzed for several criteria. In sensitivity analysis, the impact on the amount of entities is determined for any change of the parameter's magnitude. In equilibrium analysis, parameter values are identified for which the entities meet a steady state, meaning their amount does not change over time; for example, in a tumor-free equilibrium, the tumor is kept under control by the immune system. Threshold criteria are defined at which the behavior of the system changes from one state to the other. In bifurcation analysis, the solution space is scanned for discontinuous parts. Using this tool, the point can be found where one parameter changes the behavior of the system all of a sudden, jumping from one state to another; for example, below the bifurcation point, the patient remains tumor-free and above it, he develops a growing tumor.

In tumor immunology, several different problems have been addressed using differential equations systems. The simplest form consists of ordinary differential equations (ODEs) that can be solved analytically to find maxima and minima, for example, the maximal survival probability of the patient.

### 3.1. Ordinary Differential Equations to Find Generic Principles

Some of the mathematical models describe the tumor-immune interaction generally to find common mechanisms. A generic model of the influence of cytotoxic T-cells is presented by Kuznetsov et al. [29] which helps to explain the phenomena of tumor dormancy and sneaking through in a mathematical way. Leon et al. [30] explore the impact of regulatory CD25 CD4 T-cells on cancer. They propose two alternative modes of unbounded tumor growth. Either the tumor induces the production of effector T-cells that outcompete regulatory T-cells but are not able to eradicate the tumor, or a balanced expansion of both effector and regulatory T-cells is induced by the tumor, which prevents it from being destroyed by the immune cells.

The different roles of NK cells and CD8 T-cells in tumor suppression were investigated in another study [27]. The authors highlight the importance of CD8 T-cells in tumor eradication and suggest that immunotherapy should focus on the increase of their activity.

### 3.2. Ordinary Differential Equations in Specialized Therapy

The effect of innovative new cancer therapies can be estimated using differential equations systems. The influence of the newly characterized IL-21 in cancer immunotherapy was explored by Cappuccio et al. [31]. This interleukin has a role in the transition from innate immunity to adaptive immunity, and thus the authors suggest that lower doses of IL-21 should be used for low immunogenic tumors and higher doses for highly immunogenic ones. In addition, they find that cytokine gene therapy is more promising than hydrodynamics-based gene delivery.

Bunimovich-Mendrazitsky et al. [32, 33] focus on a more specific type of cancer and explore the effect of pulsed and continuous immunotherapy with Bacillus Calmette-Guérin—an attenuated strain of Mycobacterium bovis—to treat superficial bladder cancer. They calculate the amount of bacterial solution by which the tumor is eradicated but only little side effects are induced.

Kronik et al. [34] investigate the dynamics of Glioblastoma, a highly aggressive primary brain tumor, treated with *ex vivo* activated cytotoxic T-cells. Model analysis suggests that tumor eradication requires a 20-fold higher dose than had been administered in clinical studies.

Another role of the immune system in tumor formation is concerned in the cancer treatment using an oncolytic virus. In this case, the immune response against the host is considered to be negligible, while the immune cells can destroy the virus, before it is able to enter the tumor cell. To find the optimal virus administration circumventing a strong immune response against the virus, Wein et al. [35] considered different sites of virus application. They conclude that injections should be distributed equally within a solid tumor; core or rim injections alone will eventually result in tumor escape. Tao and Guo [36] extend this model, focusing on the diffusion of the virus and the immune cells and narrow down possible forms of optimal treatment.

### 3.3. Ordinary Differential Equations in Molecular Detail

A differential equations system can also focus on a specific molecular interaction. Accordingly, the importance of the Fas/FasL system was emphasized by Webb et al. [37]. The Fas ligand (FasL) can induce apoptosis in the target cell expressing the Fas receptor, while both ligand and receptor are expressed on tumor cells and T-cells at different levels depending on developmental state. The model shows that tumor regression could be enhanced by upregulated Fas receptor expression in tumor cells, but an even greater success would be gained by constitutive FasL expression in activated T-cells. This has an implication for the clinical use of broad spectrum matrix metalloproteinase (MMP) inhibitors as antiangiogenic agents. In the model, MMP inactivation results in increased transmembrane FasL and leads to a higher rate of Fas-mediated apoptosis in lymphocytes than in tumor cells; therefore, MMP treatment might be counterproductive.

### 3.4. Principle of Optimal Control

In the principle of optimal control, a control function is established that quantifies the wanted and unwanted impact of the parameters on the outcome of the model. The control function is minimized to find the desired solution. This method is based on the work of Kacser and Burns [38] and Heinrich and Rapoport [39]. A typical application nowadays is to find the best vaccination strategy with the lowest vaccine burden that is still able to eliminate the tumor. Exploring this question in human patients would be very labor intensive or almost impossible, but can easily be done using differential equations.

De Pilis et al. [40, 41] examine the effect of immunotherapy and chemotherapy on cancer growth and find that each therapy alone is not able to control the tumor; therefore, they recommend combination therapy. Also, IL-2 and adoptive cellular immunotherapy are compared in another study (Kirschner 1998), and the combination of both is able to reduce the tumor and has the least risk of inducing autoimmunity. 

Castiglione and Piccoli [42, 43] apply the principle of optimal control to determine an exact vaccination schedule in immunotherapy with autologous dendritic cell transfection. For that purpose, they build a cost function summing up the burden of the tumor and of all side effects of the immune therapy depending on the vaccination schedule and then minimizing that function to receive the optimal schedule. They recommend a vaccination schedule with one high-dose injection at the beginning of the treatment, and the other injections being smaller dosages distributed almost equally over the rest of the six months treatment period [43].

### 3.5. Delay Differential Equations

Differential equations can be enhanced to become delay differential equations (DDEs) that can account for time consumption in processes like cell division or other specific behavior. Delayed feedback and oscillatory behavior can be efficiently described using this method. For instance, Kim et al. [44] study the dynamics of chronic myelogenous leukemia (CML) under imatinib treatment including the influence of immune cells. They use delay differential equations, whereby the delay term is used to incorporate the time for cell division. The model suggests a combination of immunotherapy and imatinib treatment to optimally sustain the antileukemia T-cell response. Another study elucidates the effect of immunotherapy in leukemia patients after bone marrow transplantation to study specifically the graft-versus-leukemia effect [45]. A delay term in the differential equations system is used to account for the progression of cells through different modes of behavior. The authors conclude that high concentrations of donor T-cells slightly favor tumor elimination, but also increase the risk of graft-versus-host disease. Interestingly, higher initial concentrations of general host blood cells enhance the success rate more significantly whilst also avoiding the risk of graft-versus-host disease. This result can be applied directly to clinical treatment.

### 3.6. Partial Differential Equations

Partial differential equations (PDEs) are the most advanced form of differential equations; hence, they are also most demanding mathematically. PDEs are commonly used either to account for the progression of cells through a developmental process (age-structured model) or to model spatiality (spatiotemporal model). Matzavinos et al. [46] make use of PDEs to study the geometry of a tumor interacting with tumor-infiltrating cytotoxic lymphocytes (TICLs). This approach focuses on the motility of TICLs that can move at random or towards increasing chemokine concentration inside the tumor. The mechanism elucidated in this work may help to explain the phenomenon of tumor dormancy. A similar approach [13] tries to illuminate the growth pattern of a solid tumor depending on the attack of tumor-associated macrophages and their movement.

PDEs can also be used to model the movement of tumor cells, as in the study of Eikenberry et al. [47], where melanoma invasion into healthy tissue was simulated. The authors observe that immune cells can have opposed effects as they can both destroy tumors or can induce tumorigenic expansion through the production of angiogenic factors.

## 4. Rule-Based Modeling

**Figure 3:**
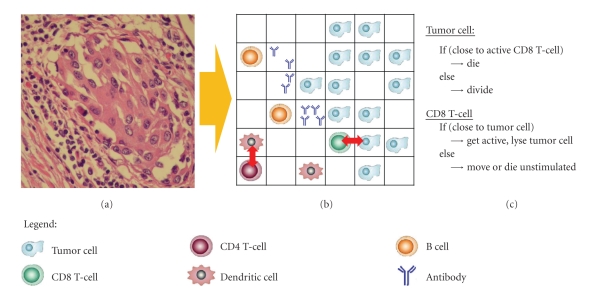
Agent-based modeling. (a) Lymphocytes infiltrating an urothelial carcinoma of the bladder. (b) Translation to an ABM. Cells move and interact in a grid. Each cell can occupy one grid space, while antibodies or cytokines have a continuous concentration at each grid space. (c) A simplified part of the underlying rules for the agents is shown.

In immunology, the two main simulation approaches in rule-based modeling are Agent-Based Models (ABMs) and Cellular Automata (CA), which are closely related. In ABMs, discrete autonomous units or agents interact with each other at discrete time steps following a set of logical rules, depending on the state of their environment. The agents are identifiable, and their environment is represented by a grid. They are able to adapt to changes in their surroundings and, therefore, need some sort of memory. A simplified ABM is shown in Figure 3, illustrating how observed phenomena are translated into behavioral rules for the entities in a grid.

**Figure 4:**
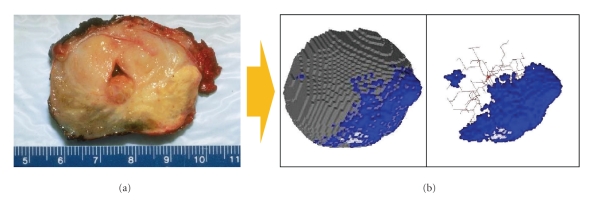
Cellular Automata. (a) Human prostate gland containing carcinoma at the lower right part, seen as a yellowish mass [23]. (b) Cellular automata named CancerSim [49] simulating tumor growth. Tumor cells are shown in blue, healthy tissue in gray, and blood vessels in red, dual view with and without healthy tissue. The three-dimensional shape of the prostate gland and the simulated structure can be compared to improve the model and to explain the observed phenomena.

CA are closely related to ABMs even though there are some important differences. In ABMs, agents are mobile whereas in CA they have fixed positions. Updating of the agents' state is usually performed in a synchronous way in CA while this is not always the case in ABMs. The understanding of the environment in ABMs also differs from the one in CA. In ABMs, it is discretized into micro-compartments which can hold a variety of information whereas the environment of agents in CA is described by the von Neumann or Moore neighborhood which consider four or eight neighbors for each agent, respectively [48]. Another aspect is that CA rule sets mostly comprise strictly deterministic rules, and ABMs often include a mixture of stochastic and deterministic elements. An example of CA is shown in Figure 4, where a prostate tumor is reconstructed using a CA called CancerSim [49]. The three-dimensional visualization of CancerSim can be compared to the observed tumor and the model can simulate the progression of cancer.

It has to be kept in mind that the modeling approaches, ABMs and CA, are very similar to each other as they are both rule-based, and it is not unusual to find hybrid forms in which elements from both methods are used. 

In immunology, rule-based models are particularly useful because cells and molecules are modeled as individual agents that may have specific ligands or receptors on their surface. An additional advantage is the possibility of including the three-dimensional space explicitly in the simulation, where each cell can be exactly located and change its activation state depending on its direct environment. This way, complex patterns evolve from a set of simple behavioral rules. 

### 4.1. Tumor-Immune System Interaction in Rule-Based Modeling

When simulating the immune response to tumor formation, the use of discrete modeling techniques as CA or ABMs is advantageous due to the possibility of considering the activity of individual cells and their interactions. The idea of simulating the immune system using discrete automata was first introduced by Kaufman et al. [50]. In their model, Boolean values are used to represent different cellular populations whereas the interactions between each other are defined by simple rules.

In early approaches to simulating tumor-immune interactions, each automaton describes the concentration of one cell type [51]. Discrete two-state variables are used to specify the concentration of particular cell types (high or low) and the functionality of their epitopes (is recognized, is not recognized). In this model, the killer role of macrophages as well as the difference between antigen recognition by T- or B-cells is ignored; still, the model captures many crucial aspects of the immune response to tumor formation.

Agent-based modeling proves to be particularly useful for modeling the early stages of tumor growth before solid tumor or metastasis formation, because in this phase of tumor development, it is necessary to consider the activity of each single cell. A model dealing with the interaction of the immune system with a tumor at this stage was presented by Mallet and de Pillis [52]. In this model, a cluster of tumor cells is studied which are supplied with nutrients through a blood vessel. The dependence of different tumor morphologies such as spherical and papillary or lymphocyte-infiltrated growth on several key model parameters related to the interplay between the immune system and the tumor is shown. For this purpose, a hybrid modeling approach is used. Cells' behavior is described by a set of probabilistic rules whereas chemical diffusion is simulated via deterministic PDEs. The simulation comprises NK cells and cytotoxic T-lymphocytes and considers their spatiotemporal interaction with normal and tumor cells.

### 4.2. Rule-Based Modeling in Immune Therapy

Recently, immune therapy of tumors is becoming increasingly well established [55, 56]. Nevertheless, several questions concerning the dosage, vaccination schedules, usage of carriers, and so forth are still open. Answering these questions experimentally remains difficult due to the high number of experiments required, which result in high costs and are very time consuming. Simulations can be extremely helpful in order to predict optimal therapy strategies and reduce the amount of experiments necessary. Several attempts to answer these questions have been performed by different groups based on a former ABM of the immune system called IMMSIM [57].

Among them, Castiglione et al. developed an elaborated model of the immune response to tumor antigens [58]. The model includes several behavioral patterns of the immune system: hematopoesis, antigen digestion and presentation by B-lymphocytes, macrophages and dendritic cells, the hypermutation of antibodies, and last but not least, cytotoxicity by CD8 T-cells. In order to simulate the immune recognition, epitopes, and peptides are represented by binary strings, and their immunogenicity is defined by the hamming distance, which is the number of complementary bits in bit-wise comparison. The aim of this work is to evaluate the effects of repeated injections of tumor-associated antigen (TAA) together with carriers on the humoral as well as on the cellular immune response. The authors find that the administration of TAA with multiple carriers causes the strongest immune response against the tumor. The administration of TAA with one single carrier fails because a stronger immune response against the carrier and not the TAA takes place. 

ABMs are also used to describe tumor vaccination in mice with the Triplex vaccine. The model SimTriplex is an ABM that describes the relevant processes of the competition between mammary carcinoma and the immune system [59, 60]. Its results show a strong correlation with experimental results. This modeling framework is also based on IMMSIM [57]. In a further approach, the model is used to optimize vaccination schedules by the use of a genetic algorithm to drive the simulator [61] and later by a simulated annealing approach [62]. This makes it possible to use the model as a virtual mouse with which extensive *in silico* experiments can be performed.

### 4.3. Virotherapy Simulated by Agent-Based Modeling

Another approach in tumor therapy is to attack the cancer with oncolytic viruses, which are capable of killing cancer cells or inducing an immune response against them. Agent-based modeling is especially suitable to simulate oncolytic virotherapy due to the possibility of describing the interplay between cancer cells, viruses, and the immune system individually and in a multiscale way. The authors of this work [63], construct a hybrid ABM-PDE model which illustrates virotherapy in a stage of avascular tumor growth. The multiscale dynamics of tumor growth are defined by probabilistic CA rules whereas the dynamics of nutrients and viruses are described by reaction-diffusion equations. The modeling result suggests that for a successful single-agent virotherapy, the host immune system must be strongly inhibited, and a potent virus with high intratumoral mobility is to be used.

## 5. Application of Modeling Results

**Figure 5:**
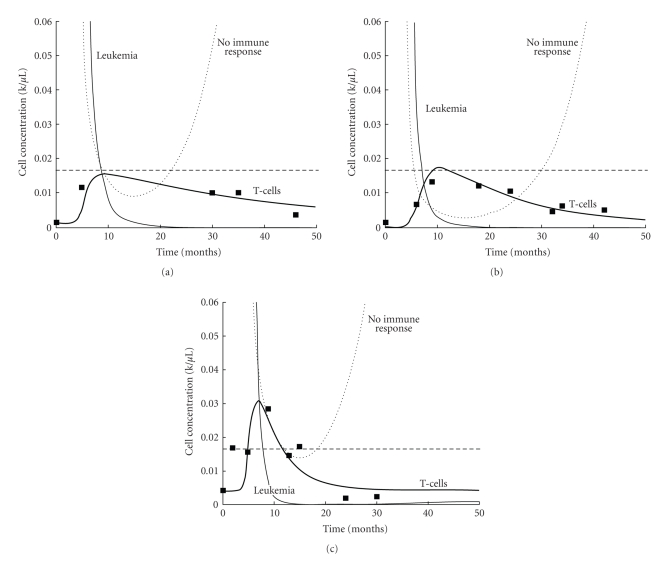
Differential equations model validation with experimental data of chronic myelogenous leukemia treatment with imatinib [44]. (a), (b), and (c) show data from different patients along with simulated results using individual parameters for each of the patients. Simulated cell concentrations are compared to measurements of T-cell numbers from [53] (cell numbers are scaled down by 2500 to show relative magnitudes). Simulated curves are shown as lines and experimental data as black squares; the horizontal dashed line indicates the approximate level of complete cytogenetic remission. ‘‘No immune response” corresponds to the predicted tumor cell number using a model that does not include the immune response [54], ‘‘Leukemia” corresponds to the results of another model that takes the immune response into account [44]. The ‘‘T cells” curve is obtained with the model by Kim et al. and is compared to experimentally observed T-cell numbers from different patients [53].

The most elaborate modeling approach is futile if it cannot be applied to reality. Therefore, it is essential to compare simulation results of a model to experimental data from the laboratory or the clinic. As an example, a comparison of the model of Kim et al. [44] to clinical data is shown in Figure 5. The parameters of three different patients (Figures 5(a), 5(b), and 5(c)) are incorporated into this model simulating the immune response to Chronic Myelogenous Leukemia under imatinib treatment. Clinically observed T-cell numbers [53] are compared to the simulated curve that represents the data points sufficiently. Based on several of these tests, the model is used to simulate the course of disease shown in the “Leukemia” curve and is compared to a similar model prediction that does not account for immune reaction (“No immune response”). 

After the validity of a model is proven using experimental data, it can be used to predict a clinical outcome, to improve a certain treatment, or to elucidate unknown mechanisms. Through collaboration with wet-lab researchers, it is possible to achieve an optimal interplay in which both sides profit from the knowledge gained from the analyzed system. Cheng et al. [64] describe the fruitful cooperation between his group and a collaborating wet-lab group, which gained important insights into the dynamics of the memory T-cell responses under sequences of heterologous viral infections. The mathematical model (IMMSIM) not only could simulate the biological findings but also could predict the experimental outcome correctly. From this collaboration, it could be shown that long-term memory loss is accounted for by active attrition by virus-induced type 1 interferon and not by the competition between memory cells.

## 6. Advantages and Disadvantages of the Modeling Types

The advantages of theoretical models over experimental work and clinical studies are obvious, mathematical and computational techniques are by far less expensive, less time consuming, and it is possible to change environmental influences and parameter scales easily.

As presented, both continuous differential equations systems and discrete ABMs or CA have been applied successfully to tumor immunology, each having its own advantages and disadvantages. 

Differential equations are easier to analyze, parameter sensitivity is measurable, and the solution space can be determined. It is also more straightforward to adjust global parameters and fit the model to experimental data using differential equations. On the other hand, differential equations systems are mainly limited to a specific observable phenomenon, and it is nearly impossible to capture the whole complexity of a biological system. By contrast, rule-based models can deal with a lot of different entities and can be easily extended with new insights from experimental research. In addition, rule-based models reproduce complex patterns from simple behavioral rules. However, rule-based models are difficult to analyze in terms of parameter sensitivity and solution space. Furthermore, most rule-based models are not completely deterministic, but they include stochastic elements which complicate the analysis additionally. The same holds true for the computational efficiency; differential equations systems are not too computationally demanding, while rule-based models might be limited by computational capacity.

The great advantage of rule-based models is their capability of distinguishing every single cell or molecule in its location, developmental state, and specificity. Using differential equations, one is limited to homogeneous populations that might not correctly represent immune cells with their specific receptors. 

After all, the choice of the modeling technique always depends on the question of interest. If the advantages of both modeling approaches are desired, the newly emerging hybrid models might be favored. In hybrid models, the underlying architecture of an ABM is extended with differential equations to simulate continuous parts of the system, as for example the interaction strength between two cells with matching receptors.

## 7. Outlook

Theoretical models are established tools in medical science and support experimental work in various ways. Their major drawback is that models can only be as good as the data or the theory they are based on, and every result has to be verified experimentally. Several parameters have to be estimated as they are not known or are not even accessible from experiments. Theoretical models can focus on main mechanisms leaving out perturbing environmental effects. Thus, they can be used to support or to contradict a theory or to search for optimal conditions for a desired outcome. One application is to improve individualized medicine as parameters of a theoretical model can be easily adjusted to the requirements of a specific patient, and the individual optimal treatment schedule can be gained.

Certainly, theoretical models and applied immunology will grow hand in hand, as experimental data is needed to establish theoretical models, and the results from simulation can help in a more efficient design of experiments.

## Figures and Tables

**Table 1:** Examples of possible data sources for model building and refinement.

Experimental results from the literature or own laboratory	Databases
Experimental results:	General databases:
(i) Cell division rates	(i) KEGG [17] (http://www.genome.jp/kegg/)
(ii) Expression data	(ii) Reactome [18] (http://www.reactome.org/)
(iii) Growth curves	Immunological databases:
(iv) Binding affinities	(i) AntiJen [21] (http://www.darrenflower.info/antijen/)
(v) Diffusion coefficients	(ii) IEDB [19] (http://www.immuneepitope.org/)
	(iii) InnateDB [22] (http://www.innatedb.org/)

## References

[1] Kitano H (2002). Systems biology: a brief overview. *Science*.

[2] Synnergren J, Olsson B, Gamalielsson J (2009). Classification of information fusion methods in systems biology. *In Silico Biology*.

[3] Kitano H (2002). Computational systems biology. *Nature*.

[4] Mantovani A, Allavena P, Sica A, Balkwill F (2008). Cancer-related inflammation. *Nature*.

[5] de Visser KE, Eichten A, Coussens LM (2006). Paradoxical roles of the immune system during cancer development. *Nature Reviews Cancer*.

[6] Rosenberg SA (2001). Progress in human tumour immunology and immunotherapy. *Nature*.

[7] Kim-Schulze S, Taback B, Kaufman HL (2007). Cytokine therapy for cancer. *Surgical Oncology Clinics of North America*.

[8] Parish CR (2003). Cancer immunotherapy: the past, the present and the future. *Immunology and Cell Biology*.

[9] Feyerabend S, Stevanovic S, Gouttefangeas C (2009). Novel multi-peptide vaccination in Hla-A2+ hormone sensitive patients with biochemical relapse of prostate cancer. *Prostate*.

[10] Van Poppel H, Joniau S, Van Gool SW (2009). Vaccine therapy in patients with renal cell carcinoma. *European Urology*.

[11] Weiner LM, Surana R, Wang S (2010). Monoclonal antibodies: versatile platforms for cancer immunotherapy. *Nature Reviews Immunology*.

[12] Koido S, Hara E, Homma S (2009). Cancer vaccine by fusions of dendritic and cancer cells. *Clinical and Developmental Immunology*.

[13] Kruse CA, Cepeda L, Owens B, Johnson SD, Stears J, Lillehei KO (1997). Treatment of recurrent glioma with intracavitary alloreactive cytotoxic T lymphocytes and interleukin-2. *Cancer Immunology Immunotherapy*.

[14] Lin A, Schildknecht A, Nguyen LT, Ohashi PS (2010). Dendritic cells integrate signals from the tumor microenvironment to modulate immunity and tumor growth. *Immunology Letters*.

[15] Kirkali Z, Tüzel E (2009). Systemic therapy of kidney cancer: tyrosine kinase inhibitors antiagiogenesis or IL-2?. *Future Oncology*.

[16] McDermott DF (2009). Immunotherapy of metastatic renal cell carcinoma. *Cancer*.

[17] Kanehisa M, Goto S, Furumichi M, Tanabe M, Hirakawa M (2009). KEGG for representation and analysis of molecular networks involving diseases and drugs. *Nucleic Acids Research*.

[18] Matthews L, Gopinath G, Gillespie M (2009). Reactome knowledgebase of human biological pathways and processes. *Nucleic Acids Research*.

[19] Vita R, Zarebski L, Greenbaum JA (2009). The immune epitope database 2.0. *Nucleic Acids Research*.

[20] Ashyraliyev M, Fomekong-Nanfack Y, Kaandorp JA, Blom JG (2009). Systems biology: parameter estimation for biochemical models. *The FEBS Journal*.

[21] Toseland CP, Clayton DJ, McSparron H (2005). AntiJen: a quantitative immunology database integrating functional, thermodynamic, kinetic, biophysical, and cellular data. *Immunome Research*.

[22] Lynn DJ, Winsor GL, Chan C (2008). InnateDB: facilitating systems-level analyses of the mammalian innate immune response. *Molecular Systems Biology*.

[23] Lipniacki T, Paszek P, Brasier AR, Luxon B, Kimmel M (2004). Mathematical model of NF-*κ*B regulatory module. *Journal of Theoretical Biology*.

[24] Lipniacki T, Puszynski K, Paszek P, Brasier AR, Kimmel M (2007). Single TNF*α* trimers mediating NF-*κ*B activation: stochastic robustness of NF-*κ*B signaling. *BMC Bioinformatics*.

[25] Tay S, Hughey JJ, Lee TK, Lipniacki T, Quake SR, Covert MW (2010). Single-cell NF-B dynamics reveal digital activation and analogue information processing. *Nature*.

[26] Helms V (2008). *Principles of Computational Cell Biology*.

[27] de Pillis LG, Radunskaya AE, Wiseman CL (2005). A validated mathematical model of cell-mediated immune response to tumor growth. *Cancer Research*.

[28] Diefenbach A, Jensen ER, Jamieson AM, Raulet DH (2001). Rae1 and H60 ligands of the NKG2D receptor stimulate tumour immunity. *Nature*.

[29] Kuznetsov VA, Makalkin IA, Taylor MA, Perelson AS (1994). Nonlinear dynamics of immunogenic tumors: parameter estimation and global bifurcation analysis. *Bulletin of Mathematical Biology*.

[30] Leon K, Garcia K, Carneiro J, Lage A (2007). How regulatory CD25+CD4+T cells impinge on tumor immunobiology? On the existence of two alternative dynamical classes of tumors. *Journal of Theoretical Biology*.

[31] Cappuccio A, Elishmereni M, Agur Z (2006). Cancer immunotherapy by interleukin-21: potential treatment strategies evaluated in a mathematical model. *Cancer Research*.

[32] Bunimovich-Mendrazitsky S, Byrne H, Stone L (2008). Mathematical model of pulsed immunotherapy for superficial bladder cancer. *Bulletin of Mathematical Biology*.

[33] Bunimovich-Mendrazitsky S, Shochat E, Stone L (2007). Mathematical model of BCG immunotherapy in superficial bladder cancer. *Bulletin of Mathematical Biology*.

[34] Kronik N, Kogan Y, Vainstein V, Agur Z (2008). Improving alloreactive CTL immunotherapy for malignant gliomas using a simulation model of their interactive dynamics. *Cancer Immunology, Immunotherapy*.

[35] Wein LM, Wu JT, Kirn DH (2003). Validation and analysis of a mathematical model of a replication-competent oncolytic virus for cancer treatment: implications for virus design and delivery. *Cancer Research*.

[36] Tao Y, Guo Q (2005). The competitive dynamics between tumor cells, a replication-competent virus and an immune response. *Journal of Mathematical Biology*.

[37] Webb SD, Sherratt JA, Fish RG (2002). Cells behaving badly: a theoretical model for the Fas/FasL system in tumour immunology. *Mathematical Biosciences*.

[38] Kacser H, Burns JA (1973). The control of flux. *Symposia of the Society for Experimental Biology*.

[39] Heinrich R, Rapoport TA (1974). A linear steady state treatment of enzymatic chains: general properties, control and effector strength. *European Journal of Biochemistry*.

[40] de Pillis LG (2007). Chemotherapy for tumors: an analysis of the dynamics and a study of quadratic and linear optimal controls. *Mathematical Biosciences*.

[41] de Pillis LG, Gu W, Radunskaya AE (2006). Mixed immunotherapy and chemotherapy of tumors: modeling, applications and biological interpretations. *Journal of Theoretical Biology*.

[42] Castiglione F, Piccoli B (2006). Optimal control in a model of dendritic cell transfection cancer immunotherapy. *Bulletin of Mathematical Biology*.

[43] Castiglione F, Piccoli B (2007). Cancer immunotherapy, mathematical modeling and optimal control. *Journal of Theoretical Biology*.

[44] Kim PS, Lee PP, Levy D (2008). Dynamics and potential impact of the immune response to chronic myelogenous leukemia. *PLoS Computational Biology*.

[45] DeConde R, Kim PS, Levy D, Lee PP (2005). Post-transplantation dynamics of the immune response to chronic myelogenous leukemia. *Journal of Theoretical Biology*.

[46] Matzavinos A, Chaplain MAJ, Kuznetsov VA (2004). Mathematical modelling of the spatio-temporal response of cytotoxic T-lymphocytes to a solid tumour. *Mathematical Medicine and Biology*.

[47] Eikenberry S, Thalhauser C, Kuang Y (2009). Tumor-immune interaction, surgical treatment, and cancer recurrence in a mathematical model of melanoma. *PLoS Computational Biology*.

[48] Gray L ( 2003). A mathematician looks at Wolfram’s new kind of science. *Notices of the American Mathematical Society*.

[49] http://www.cs.unm.edu/~forrest/software/cancersim/.

[50] Kaufman M, Urbain J, Thomas R (1985). Towards a logical analysis of the immune response. *Journal of Theoretical Biology*.

[51] Chowdhury D, Sahimi M, Stauffer D (1991). A discrete model for immune surveillance, tumor immunity and cancer. *Journal of Theoretical Biology*.

[52] Mallet DG, De Pillis LG (2006). A cellular automata model of tumor-immune system interactions. *Journal of Theoretical Biology*.

[53] Higham EM, Shen C-H, Wittrup KD, Chen J (2010). Cutting edge: delay and reversal of T cell tolerance by intratumoral injection of antigen-loaded dendritic cells in an autochthonous tumor model. *Journal of Immunology*.

[54] Michor F, Hughes TP, Iwasa Y (2005). Dynamics of chronic myeloid leukaemia. *Nature*.

[55] Dougan M, Dranoff G (2009). Immune therapy for cancer. *Annual Review of Immunology*.

[56] Jenq RR, van den Brink MRM (2010). Allogeneic haematopoietic stem cell transplantation: individualized stem cell and immune therapy of cancer. *Nature Reviews Cancer*.

[57] Celada F, Seiden PE (1992). A computer model of cellular interactions in the immune system. *Immunology Today*.

[58] Castiglione F, Toschi F, Bernaschi M (2005). Computational modeling of the immune response to tumor antigens. *Journal of Theoretical Biology*.

[59] Motta S, Castiglione F, Lollini P, Pappalardo F (2005). Modelling vaccination schedules for a cancer immunoprevention vaccine. *Immunome Research*.

[60] Pappalardo F, Lollini P-L, Castiglione F, Motta S (2005). Modeling and simulation of cancer immunoprevention vaccine. *Bioinformatics*.

[61] Lollini P-L, Motta S, Pappalardo F (2006). Discovery of cancer vaccination protocols with a genetic algorithm driving an agent based simulator. *BMC Bioinformatics*.

[62] Pappalardo F, Pennisi M, Castiglione F, Motta S (2010). Vaccine protocols optimization: in silico experiences. *Biotechnology Advances*.

[63] Paiva LR, Binny C, Ferreira SC, Martins ML (2009). A multiscale mathematical model for oncolytic virotherapy. *Cancer Research*.

[64] Cheng Y, Ghersi D, Calcagno C, Selin LK, Puzone R, Celada F (2009). A discrete computer model of the immune system reveals competitive interactions between the humoral and cellular branch and between cross-reacting memory and naïve responses. *Vaccine*.

